# New HER2-negative breast cancer subtype responsive to anti-HER2 therapy identified

**DOI:** 10.1007/s00432-020-03144-7

**Published:** 2020-02-08

**Authors:** Ian A. MacNeil, David J. Burns, Benjamin E. Rich, Sajjad M. Soltani, Samantha Kharbush, Nicole G. Osterhaus, Brian F. Sullivan, Douglas M. Hawkins, Jodie R. Pietruska, Lance G. Laing

**Affiliations:** 1 Celcuity Inc., 16305 36th Ave N, Suite 100, Minneapolis, MN 55446 USA; 2 School of Statistics, University of Minnesota, Minneapolis, MN 55455 USA; 3 Department of Developmental, Molecular, and Chemical Biology, Tufts University, Boston, MA 02111 USA

**Keywords:** Breast cancer, HER2, Cell signaling, Xenograft, Targeted therapy

## Abstract

**Purpose:**

HER2 signaling functional activity may be important to measure in addition to HER2 protein quantification when identifying patients eligible for HER2 therapies. A HER2 Signaling Function (CELx HSF) Test for HER2-negative patients uses patient’s live tumor cells on a biosensor to identify patients with abnormally high HER2-related signaling (HSFs+) likely to respond to anti-HER2 therapies.

**Methods:**

The CELx HSF test was employed to: (1) characterize the sensitivity and specificity of the test to detect abnormal levels of HER2 signaling; (2) evaluate the inhibitory effectiveness of five different anti-HER2 therapies; (3) assess the correlation between CELx HSF test detection of abnormal HER2 signaling and response to HER2 therapy using xenograft models; and (4) confirm the prevalence of abnormal HER2 signaling amongst HER2-negative breast cancer patients (HER2−/HSFs+).

**Results:**

HER2−/HSFs+ breast cancer patient samples were identified and showed sensitivity to five approved anti-HER2 therapies. Xenograft studies using both HER2+ and HER2− cell lines confirmed that CELx HER2 signaling status better predicts HER2 inhibitor efficacy than HER2 receptor status. In a study of 114 HER2-negative breast tumor patient samples, 27 (23.7%; 95% CI = 17–32%) had abnormal HER2 signaling (HSFs+). A ROC curve constructed with this dataset projects the CELx HSF Test would have greater than 90% sensitivity and specificity to detect the HER2−/HSFs+ patient population.

**Conclusions:**

The CELx HSF test is a well-characterized functional biomarker assay capable of identifying dynamic HER2-driven signaling dysfunction in tumor cells from HER2-negative breast cancer patients. This test has demonstrated efficacy of various HER2 targeted therapies in live tumor cells from the HSFs+ population and correlated the test result to HER2 drug response in mouse xenograft studies. The proportion of HER2-negative breast cancer patients found to have abnormal HER2 signaling in a 114 patient sample study, 20–25%, is significant. A clinical trial to evaluate the efficacy of anti-HER2 therapies in this patient population is warranted.

**Electronic supplementary material:**

The online version of this article (10.1007/s00432-020-03144-7) contains supplementary material, which is available to authorized users.

## Introduction

The human epidermal growth factor receptor 2 (*HER2*) gene encodes a receptor tyrosine kinase that is amplified or overexpressed in approximately 15% of human breast cancers and is a potent driver of oncogenic transformation and breast tumorigenesis (Akiyama et al. 1986; Al-Kuraya et al. 2004; Dawood et al. 2010; Fiore et al. 1987; Muller et al. 1988; Slamon et al. 1987). Eligibility for HER2 targeted therapy is currently determined by in situ hybridization (ISH) or immunohistochemistry (IHC), which measure total HER2 DNA copy number and protein levels, respectively (Wolff et al. 2013).

Only 30–40% of ISH/IHC identified HER2+ patients achieve clinical benefit from treatment with HER2 therapies, which translates to a high false positive rate using current HER2 biomarkers for patient selection (Baselga et al. 2012; Slamon et al. 2001). Moreover, retrospective analyses of both the National Surgical Adjuvant Breast and Bowel Project (NSABP) B-31 trial and the similarly designed North Central Cancer Treatment Group (NCCTG) N9831 trial found that a subset of breast cancer patients classified by ISH/IHC as HER2− unexpectedly benefited from treatment with the anti-HER2 antibody trastuzumab (Paik et al. 2008; Perez et al. 2010). This further uncovered a significant false negative rate for current HER2 biomarkers, i.e. patients that would be excluded from treatment that may indeed respond to HER2-targeted therapy. These results reveal that overexpression or amplification of HER2 is only weakly correlated to the disease mechanism associated with HER2 signaling dysfunction.

Alternative diagnostic methods that assess patients with HER2-driven tumors and better predict their responsiveness to anti-HER2 therapies are a much-needed step toward effecting precision medicine in breast cancer. An important but underappreciated limitation of current methodologies used to select patients for treatment with anti-HER2 therapies is their inability to provide a dynamic assessment of HER2 pathway activation in a tumor biopsy sample, since the sample is fixed and analyzed at a single point in time. Current biomarkers provide no information on important determinants of RTK pathway activity, such as feedback or feed-forward signaling or receptor aggregation. Such information would require an integrated test readout capable of assessing the dynamic complexity of genomic, epigenomic, proteomic, metabolomic, regio-spatial, kinetic, temporal signaling and drug response.

We have developed a test for solid tumor cells that relies upon an impedance-based measurement of cell adhesion and morphological changes, which is well-described in the literature as intimately involved in the complexities of real time cell signaling (reviewed in Hynes (1999, 2002), Juliano (2002)). The CELx HSF Breast Test, validated in a CAP/CLIA laboratory, measures signaling dysfunction while the cells are viable by observing specific cell responses to perturbation during the most appropriate time period. The cell response causes impedance changes that are measured as a change in the electron flow around the test cells related to the cell–cell gap junction (barrier function), cell-extracellular matrix attachment density (alpha function), and changes to the cytoskeletal architecture (transmembrane capacitance). To this end, a cell impedance measurement is ideally suited to quantify events related to cell signaling (Giaever and Keese 1991; Hynes 1999; Juliano 2002).

The very large differentiation between the signaling measured in the abnormal and normal signaling patient sub-groups identified by the CELx test enables the test to achieve high specificity and sensitivity when identifying signaling dysfunction for a clinical test. Addition of a targeted therapeutic, specifically designed to disrupt the signaling dysfunction so measured during the specific time period, when applied eighteen hours prior to stimulation, further confirms the diagnosis and prognosis for likely clinical benefit. To date, ex vivo measures of whole cell activity, such as apoptosis or proliferation rates, have not yielded sufficiently differentiated sub-groups within a population to achieve the specificity required to become appropriate for clinical use. The difficulty of using apoptosis and proliferation rates as an assay endpoint reflects the challenges of using normalization of differences in proliferation rates and differences in ex vivo viability. To diagnose HER2-driven signaling dysfunction in breast cancer patients with normally expressed and non-amplified HER2, (Cheng et al. 2014; Cicenas et al. 2006; DiGiovanna et al. 2005; Hudelist et al. 2006; Kurebayashi et al. 2015; Ramic et al. 2013; Thor et al. 2000; Wulfkuhle et al. 2012) we developed the CELx HER2 Signaling Function (CELx HSF) Test, a novel assay that assesses dynamic HER2 signaling activity in live patient tumor cells. This assay uses a biosensor to detect changes in the impedance value a live cell sample generates when attached via extracellular matrix to a micro-electrode. The impedance value, defined as the cellular adhesion signal, or CAS, is dependent on the type and density of adhesion proteins on the cell surface and is regulated by cell response to specific perturbations such as the addition of receptor ligands. We have demonstrated that this assay precisely measures impedance response to functional real-time HER2-driven signaling activity (Huang et al. 2016).

Patient tumor cells are tested for HER2 signaling dysfunction by: (1) individually characterizing HER3 and HER1 signaling activity by stimulating cells separately with NRG and EGF; (2) individually characterizing the activity specific to HER2 heterodimerization with the HER3 and HER1 receptor by also contacting the cells with a HER2 inhibitor. The total contribution of HER2-specific involvement in pathway signaling is determined by measuring the change in the growth factor-induced CAS in the presence of HER2-specific dimerization inhibitor. A high ΔCAS is indicative of abnormally high HER2-involved signal (Huang et al. 2016, 2017).

Previous analysis of 34 HER2-negative breast tumor cell samples using the CELx HSF test indicated that 20.5% (95% CI 10.0–37.1) of these samples were HER2-/HSFs+ (Huang et al. 2016, 2017). Further work screening multiple HER2+ and HER2− cell lines and patient samples demonstrated that there was no significant correlation between HER2 receptor expression level and the HER2-initiated signaling activity quantified by the CELx HSF Test (Huang et al. 2017).

In this study, we evaluated the efficacy of a panel of HER2 targeted therapies in HER2−/HSFs+ breast cancer patient cell samples and HER2+ /HSFs+ breast cancer cell lines. Mouse xenograft models were used to evaluate the efficacy of a HER2 targeted therapy in HER2+ and HER2− negative cell lines to determine the correlation between a CELx HSF Test result, HER2 receptor status, and HER2 drug response in an animal model. Finally, we examined tumor tissue from an independent set of 114 individual HER2-negative breast cancer patients to determine the prevalence of abnormal HER2 signaling activity amongst the population of breast cancer patients lacking HER2 overexpression or amplification.

The findings of this study suggest that a new sub-type of HER2−/HSFs+ breast cancer identified by the CELx HSF Test may be responsive to HER2 targeted therapies. Clinical trials to test this hypothesis are in progress.

## Materials and methods

### Collection of patient specimens

Healthy and tumor specimens were obtained from excess resected human breast tissue from women over 18 years of age undergoing standard-of care therapeutic surgery and histological diagnosis. Breast tumor specimens were collected from sites with Institutional Review Board (IRB) approval to supply human tissue specimens as well as from sites that have pre-established tissue acquisition programs that obtain informed consent for provision of patient specimens independent of Celcuity. All subjects providing tissue underwent surgical excision of their breast cancer tumor per each facilities’ standard of care. Collection sites included the University of Minnesota (beginning in January 2015); members of the Cooperative Human Tissue Network (The Ohio State University Hospital System, Case Western University Hospital System, the University of Pennsylvania Hospital, The University of Alabama at Birmingham Hospital, and Vanderbilt University Medical Center, beginning in February 2015); Park Nicollet Hospital (beginning in March 2015); Aurora Health Care (beginning in May 2015); and Roswell Park Cancer Institute (beginning in June 2015). Patient data for all de-identified specimens collected included the subjects’ age, health, gender, and demographic information and a redacted pathology report with ER and HER2 biomarker detail, stage, histology, and metastasis by lymph node status. Only subjects with confirmed breast cancer (any stage, including recurrence) with identifiable tumor mass who were already scheduled for core needle biopsy, fine needle aspiration, vacuum-assisted core biopsy, image-guided core needle biopsy, surgical biopsy, or surgical resection were enrolled in the study and provided excess tissue for analysis by the CELx HSF Test. Subjects with tumor mass insufficient for the attending physician or pathologist to obtain 2–4 biopsy cores or a 3 mm × 3 mm × 1 mm tissue slice were excluded, as were subjects with a clinical history of HIV/AIDS, HBV, or HCV. Typically, 20–50 mg of excess tissue from a tumorectomy or biopsy procedure were sent to Celcuity. The entire 20–50 mg specimen analyzed was processed as a whole to ensure that each cell sample sent to Celcuity tested was representative of the starting tissue. In addition, all samples tested were comprised of zero passage cells to preserve the heterogeneity of the sample as received and to reduce the probability of genetic and epigenetic alterations that might occur over time in culture (DeRose et al. 2013). Small amounts of tissue did not allow for specific location sub-sample division and analysis in this study. At some future point, this would be of interest in the investigation of tumor heterogeneity. The tumors analyzed were obtained from a single tumor site; tissue from different tumor sites was not available. The clinical testing laboratory used for all pre-analytical cell preparation and analytical testing for all patient specimens is accredited by the College of American Pathologists (CAP) and has Clinical Laboratory Improvement Amendments certification (CLIA) to insure uniformity and quality of the processes and results.

### Establishment of primary breast tumor cell cultures

Methods for sample collection, storage, transport, tissue extraction, and primary cell culture have been previously described (Huang et al. 2016, 2017). Briefly, patient breast tumor tissue was delivered from the participating clinic to the laboratory in less than 30 h at 0–8 °C, accessioned, and then minced with scalpels to < 2-mm pieces and either used fresh or cryopreserved until further analysis (Unisol, Cell and Tissue Systems, Charleston, SC). Tumor samples were rejected for culturing/testing if they exhibited any of the following properties: highly necrotic, calcified, fibrotic, and/or acellular pathology report descriptions. Samples were cultured between 5–21 days. For CELx HSF testing, 20–40 mg of tissue was enzymatically disaggregated with collagenase and hyaluronidase (Worthington Biochemical, Lakewood, NJ) at 37 °C in 5% CO_2_. Disaggregated tissue was washed and plated on 4-well tissue culture plates in serum-free mammary epithelial cell media at 37 °C in 5% CO_2_ without passage until at least 250,000 cells were available for subsequent testing.

### Human and murine cell lines

Human breast cancer cell lines used in this study included SKBr3 (HER2+ /HSFs+), BT474 (HER2+ /HSFs−), BT483 (ER-/HER2−/HSFs+), ZR75-30 (HER2+ /HSFs+), HCC1569 (HER2+ /HSFs−), HCC1954 (ER-/HER2+ /HSFs−), HCC202 (HER2+ /HSFs+), MDA-MB361 (HER2+ /HSFs−), AU565 (HER2+ /HSFs−) (all from American Tissue Type Collection; ATCC, Manassas, VA), and EFM192A (HER2+ /HSFs+) (from Leibniz Institute DSMZ, Germany). Cell lines were maintained according to ATCC recommendations and authenticated periodically by ATCC STR profiling. All cell media were obtained from Mediatech (Manassas, VA) and fetal bovine serum (FBS) was from Hyclone (Logan, UT).

### Flow cytometry

Flow cytometry was performed using a BD FACSCalibur (BD Biosciences, San Jose, CA) with cells harvested at the time of CELx HSF Test using methods previously described by others (Huang et al. 2016; Lim et al. 2009). Data were analyzed with FlowJo 2 software (version 10.4) (FlowJo, Ashland, OR). Antibodies used are listed in Supplemental Table 3.

### CELx HER2 signaling function (HSF) test

To provide a continuous, real-time assessment of HER2 dynamic signaling activity, the CELx HSF Test was performed as described elsewhere (Huang et al. 2016, 2017). In brief, 12,750 primary cells were seeded in serum-free base mammary epithelial cell medium in a row (12 wells) of an xCELLigence Real-time Cell Analyzer (RTCA) 96-well Microplate and Bioanalyzer (ACEA Biosciences, San Diego, CA, USA). Electrode impedance caused by the presence of viable adherent cells on the biosensor plate surface (the cell attachment signal, or CAS) was measured, recorded, and calculated continuously over the course of each experiment as a quantitative result. The test score was calculated as the sum of the differences in CAS (ΔCAS) between the cells treated with growth factor agonist (3 nM NRG or 0.3 nM EGF) in the absence and presence of the HER2 specific antagonist (10 µg/mL 2C4 mAb). This data were then transformed into a final qualitative test result to characterize the activity level of the HER2 signaling pathway function in the tested patient tumor cells. Sample results were normal (HSFs-) or abnormal (HSFs+) for HER2 signaling function based on a cutoff value previously determined (Huang et al. 2016) and validated in this study. Final growth factor and 2C4 reagent concentrations used in the final test format were selected from titration curves for multiple cell samples to represent the EC or IC_90_ for the larger population (Huang et al. 2016).

Studies determining the nature and comparative extent of HER2-mediated inhibition of HER2-negative primary tumor cell samples and HER2+ cell lines used a panel of four HER2 targeted antagonists at clinically relevant concentrations (pertuzumab 10 μg/mL, lapatinib 200 nM, neratinib 500 nM, and afatinib 120 nM), in conjunction with 3 nM NRG1, a specific agonist of hetero- or homo-HER3 dimers. Impedance signals with or without antagonist (drug) were measured and quantified, and the percentage inhibition per sample [(CAS_NRG−_CAS_NRG+drug_)/CAS_NRG_ × 100] was determined from the average ΔCAS of duplicate well results. Average inhibition was calculated for all cell lines (*n* = 9) and tumor cell samples (*n* = 7). Antibody inhibition studies used pertuzumab 10 μg/mL and trastuzumab 10 μg/mL alone, or in combination, and inhibition averages were estimated for all cell lines (*n* = 4) and tumor samples (*n* = 5).

EGF and NRG1 (R&D Systems, Minneapolis, MN, USA) were freshly prepared in assay medium at an 11× stock concentration (0.33 nM and 3.3 nM, respectively) and added 24 h after cell seeding. HER2 inhibitors were freshly prepared in serum-free, stimulant-free, mammary epithelial cell medium at 11× stock concentrations and added to the sensor plates at least 18 h prior to the addition of growth factors. 2C4 (a HER2 receptor dimerization blocking mouse monoclonal antibody) was provided from the Dana-Farber Cancer Institute Monoclonal Antibody Core production facility (Boston, MA, USA). Lapatinib, neratinib, and afatinib, were purchased from SelleckChem (Houston, TX, USA). Trastuzumab and pertuzumab were obtained from Kronan Pharmacy (Uppsala, Sweden). All growth factors and inhibitors were dispensed with a VIAFLO automatic liquid handler (Integra Biosciences).

### Mouse xenograft studies

All animal studies were performed according to the guidelines of the Institutional Animal Care and Use Committee of the University of Minnesota. Two cell lines developed from primary breast tumors, BT-483 (ER^+^/HER2^−^/HSFs+) and HCC1954 (ER^−^/HER2^+^/HSFs-), were maintained in RPMI-1640 medium supplemented with 10% fetal bovine serum until harvesting for in vivo studies. For all xenograft experiments, 2 × 10^6^ cells were suspended in unsupplemented RPMI-1640 medium and Matrigel (BD#356237, Corning, New York, USA, growth factor reduced, classic formulation) in a 1:1 ratio and injected into the second right mammary fat pad of healthy, naïve, six-week old, 20 g average body weight, female NOD.Cg-*Prkdc*^*scid*^* Il2rg*^*tm1Wjl*^/SzJ (NSG) mice (The Jackson Laboratory, Bar Harbor, ME) in a 150-μL injection volume. Animals were housed as five individuals/cage. 17-β estradiol was administered via subcutaneously implanted pellets (17 mg, 60-day release; Innovative Research of America, Sarasota, FL, catalog #SE-121) one day prior to tumor cell injection. When the average size of tumors in all mice was 150 mm^3^ (with a minimum tumor size of 100 mm^3^), mice were randomized to receive 75 mg/kg lapatinib daily (*n* = 10 per cell line) or vehicle control (10% Captisol; Ligand, San Diego, CA, USA; *n* = 10 per cell line) by oral gavage. Lapatinib treatment of mice started from Day 34 and lasted for 19 days (53 days after tumor cell implantation). Animals were assigned to treatment groups using a restricted randomization strategy to assign mice to the treatment or control group while balancing average tumor volume in each group at the beginning of treatment. Mice were monitored for weight loss, and mice experiencing greater than 20% weight loss were euthanized. Tumor measurements were recorded with calibrated digital calipers every three to four days (twice per week) until drug treatment was initiated; thereafter, tumors were measured at least every other day. Mean tumor volume was calculated using the formula: volume = ((width)^2^ × length)/2.

### Statistics

The population sample size calculation for this study consists of establishing the proportion of the HER2-negative population that exhibits abnormal HER2 signaling, with a target of 95% margin of error that is not greater than 50% of the estimate. The expectation was that 15% to 25% (average 20%) of the population will be abnormal, leading to required sample sizes of 48 at the low end and 91 at the high end. A sample size of 114 was used to reduce the margin of error. The actual margin of error was 7.16%. Pearson correlation analysis was performed with GraphPad Prism 6 to evaluate the relationships among the variables of interest in the different experiments. All dose–response curves were obtained using nonlinear regression curve fitting with GraphPad Prism 6. Analysis of the xenograft data was performed using *t* test with a 95% CI (α = 0.05). Statistical analysis of the CELx HSF Test results for HER2-negative patient tumor samples to establish signaling cutoff was performed using the normalmixEM procedure in the R statistical analysis package mixtools (https://www.r-project.org/) with normal population distribution assumptions, following initial distribution comparison of the 114 tumor data set with a prior 34 tumor data set using the Kolmogorov–Smirnov non-parametric two-sample test. Formal significance testing of the fit outcomes was done using the likelihood ratio test.

### Study approval

Human tissues and supporting information were de-identified prior to delivery to the clinical test laboratory. Advarra Institutional Review Board (Columbia, MD) determined that this research did not involve human subjects as defined under 45 CFR 46.102(f) and issued a written IRB exemption. The mouse study protocol was reviewed and approved by the Institutional Animal Care and Use Committee of the University of Minnesota Academic Health Center, Center for Translational Medicine.

## Results

### Patient-derived cultures of primary tumor are comprised of heterogeneous breast epithelial cells

Cell samples were derived from short-term (typically 14 days or less) culture of cells and cell clusters extracted from a small tissue specimen (typically 25 mg). While the CELx HSF test readout is not a measure of cell viability, only viable cells are capable of adhering to the biosensor and providing a testable sample. Sample inclusion criteria excluded use of any sample that was non-viable, calcified, fibrotic, acellular, and or consisted solely of scar tissue, as ascertained by physical observation and correlated with accompanying pathology reports. Approximately 14% of all prospective specimens received were excluded based on non-viable criteria. Of tissue samples meeting the inclusion criteria, 98% yielded a viable cell sample; the two samples not yielding a viable cell sample were likely contaminated at the time of collection. Cell colonies from patient tumor tissue specimens appeared heterogeneous and phenotypically epithelial, marked by closely apposed cells with a cobblestone appearance and expressing classical epithelial cell biomarkers by flow cytometric analysis (Supplemental Fig. 1), as described previously (Huang et al. 2016; Lim et al. 2009).

### Specificity of the CELx HSF test in breast primary tumor cells

The CELx HSF Test measures HER2-related signaling in live breast cancer cells in real-time by evaluating the difference between agonist-induced (ligand/growth factor) signals in the absence or presence of a HER2 dimerization blocker (monoclonal antibody 2C4). HER2 is known to heterodimerize with HER1, HER3, and HER4 to activate agonist-dependent signaling, and the contribution of HER2 signaling function (HSF) in a given sample takes into account agonist-driven activation of the two main dimerization partners of HER2: HER1 and HER3. NRG1b (which binds HER3 and HER4), EGF (which binds HER1), and the monoclonal antibody 2C4 (a HER2 receptor dimerization blocker) were employed to demonstrate that the CELx HSF signals are specifically attributable to HER2-related signaling. Pertuzumab, which is also used in this study, is a humanized version of the mouse monoclonal 2C4 and is approved for use to treat HER2+ breast cancer patients.

**Fig. 1:**
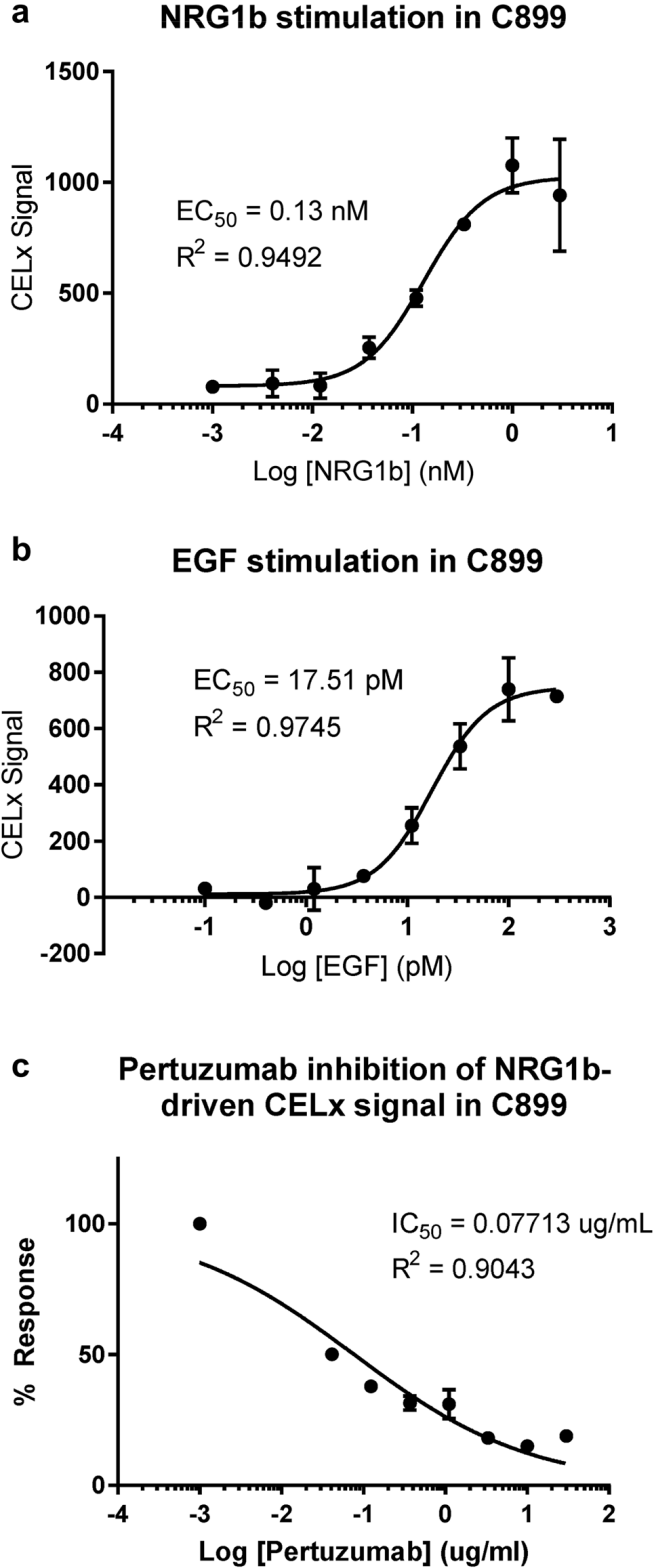
Optimization and specificity of CELx HSF Test in primary tumor cells. Dose–response curves of NRG1b (**a**) and EGF (**b**) stimulation of CELx signals in C899 patient primary breast tumor cells that were classified as HER2-negative based on histopathological analysis. **c** Dose–response curve of pertuzumab showing its specific inhibitory effect on NRG1b-driven CELx signal

The EC_50_ for each growth factor agonist, NRG1b (130 pM) and EGF (17.5 pM), (Fig. 1a, b) was established using primary cell cultures derived from a HER2-negative breast tissue specimen (C899). The growth factor concentrations used were within the physiological ranges observed in human serum (Agus et al. 2002). Signal magnitude correlated with the dose of each growth factor, and the dose response curve fit values were in close agreement with previous reports (Huang et al. 2016; Press et al. 2008).

To further define the specificity of the test signal, we next determined that growth factor-initiated CELx HSF HER2 signals could be inhibited by the 2C4 monoclonal antibody. 2C4 specifically blocks HER2 dimerization with HER1, HER3, and HER4, thereby enabling assessment of the proportions of HER2-dependent signals in the quantitation of the CELx HSF Test values (Huang et al. 2016; Press et al. 2005, 2008). 2C4 inhibited the NRG1b-initiated signal with an IC_50_ of 0.1 µg/mL (~ 0.7 nM) (Fig. 1c), and the test concentration was selected at 10 µg/mL, which was sufficient to inhibit ~ 100% of the NRG1b-induced signal in test development samples. The 10 µg/mL 2C4 is a conservative concentration that is also consistent with therapeutic levels of pertuzumab in patient sera (Perjeta (pertuzumab) [package insert] Genentech, Inc. South San Francisco, CA 2012; Quartino AL 2017).

**Fig. 2:**
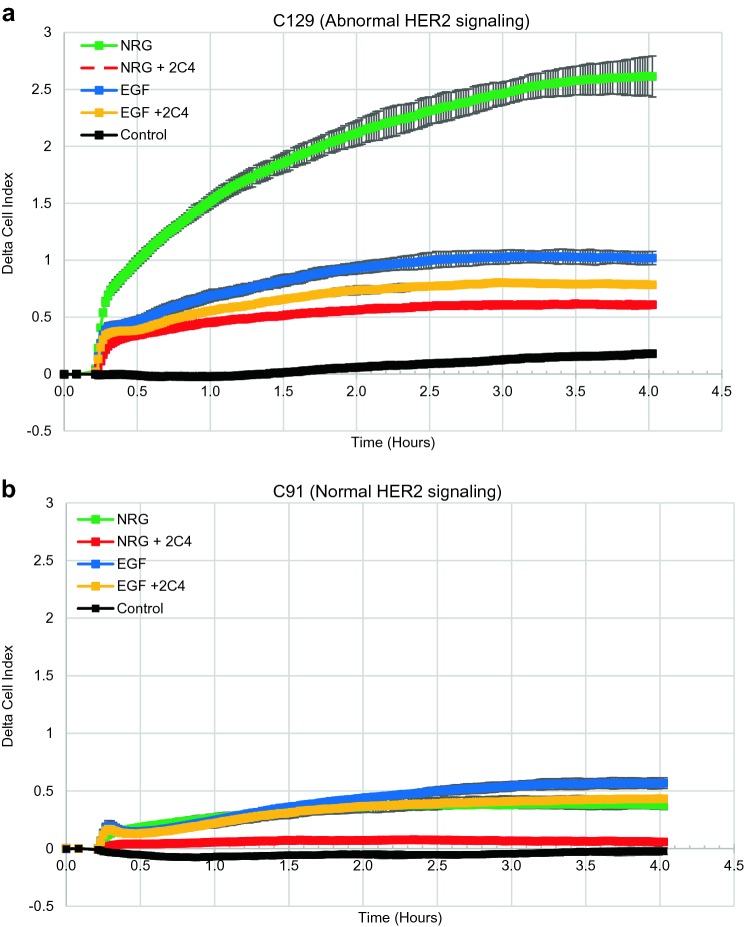
Representative CELx HSF Test tracings in primary tumor cells. **a** Time course versus change in CAS graph for a patient with abnormal HER2 signaling following addition of agonists NRG and EGF with and without receptor dimer blocker to determine HER2 participation in the agonist signal. **b** Time course versus change in CAS graph for a patient with normal HER2 signaling

The fundamental concepts of the CELx HSF test are illustrated in Fig. 2 using the above derived growth factor and drug concentrations. The ability to identify patient samples demonstrating intrinsically abnormal activation of the HER2 pathway, as indicated by responsiveness to growth factor (EGF or NRG1b) and inhibition of response by 2C4, is shown for two primary patient samples derived from HER2 negative breast tissue. Real-time tracings of impedence change over a 4-h period of growth factor-stimulation identify sample C129 as exhibiting abnormal HER2 signaling (HER2−/HSFs+ ; Fig. 2a) and sample C91 as exhibiting normal HER2 signaling (HER2−/HSFs−; Fig. 2b). Taken together, these results demonstrate that the CELx HSF Test can specifically detect growth factor-induced, HER2-related signals and determine whether a HER2-driven test signal is sensitive or insensitive to a HER2-targeted drug.

### HER2 targeted antibody therapeutics show equivalent or higher efficacy in HER2−/HSFs+ primary tumor cells compared to HER2+ /HSFs+ cell lines

**Fig. 3:**
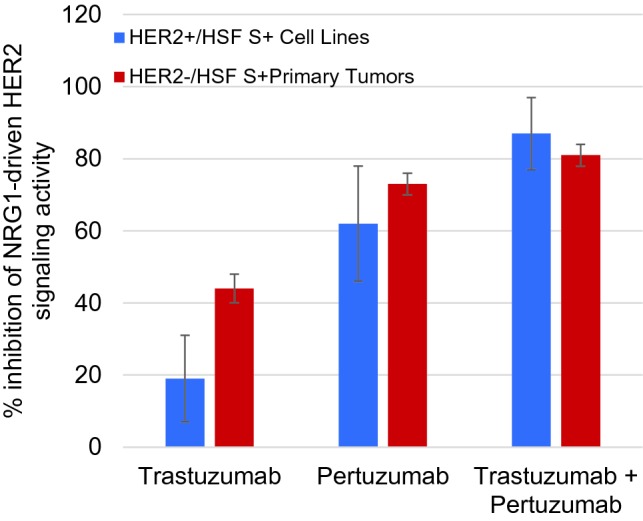
Percent inhibition of NRG1b CELx signal for HER2+ cell lines versus HER2−/HSFs+ patient samples**.** Inhibition of NRG1-driven HER2 signaling by pertuzumab (10 mg/mL) or trastuzumab (10 mg/mL) alone or in combination in the CELx test. Percentages shown represent the average of four HER2+ HSFs+ cell lines (SKBR3, EFM192A, HCC1569, and ZR75-30) or 5 HER2−/HSFs+ primary tumor samples (C978, C133, C264, C309, and C371), performed with 2 technical replicates per biological sample

Previous work has shown that the magnitudes of abnormal HSF signaling in HER2-negative primary tumor cells could reach levels as high as HER2+ /HSFs+ cell lines (Huang et al. 2016, 2017). We have expanded this work with the two current standard-of-care anti-HER2 drugs, pertuzumab and trastuzumab. Four HER2+ /HSFs+ cell lines and five HER2−/HSF_S+_ patient tumor samples were stimulated with NRG1b in the presence of therapeutic concentrations of pertuzumab, trastuzumab, or a combination of both drugs. Pertuzumab and trastuzumab alone were each more effective in the HER2-negative primary tumor cell group than in the HER2+ cell line group, consistent with previous studies using breast cancer cell lines (Agus 2002; Junttila et al. 2009), Pertuzumab inhibited NRG1b-initiated HER2 signaling by an average of 62% (range 47–83%) in HER2+ cell lines and an average of 73% (range 68–75%) in HER2-patient cell samples (Fig. 3). Trastuzumab had a smaller inhibitory effect on NRG1b-initiated HER2 signaling in HER2+ cell lines (average 19%, range 1–27%) but more effectively inhibited HER2 signaling in the HER2− patient cell samples (average 44%, range 39–48%) (Fig. 3). Trastuzumab and pertuzumab in combination inhibited NRG1b-initiated HER2 signaling in both the HER2+ cell lines (average 87%, range 77–100%) and the HER2-negative patient cell samples (average 81%, range 78–86%) to a greater extent than either drug alone, with no evidence that the drugs interfered with each other’s inhibitory activity (Fig. 3).

**Fig. 4:**
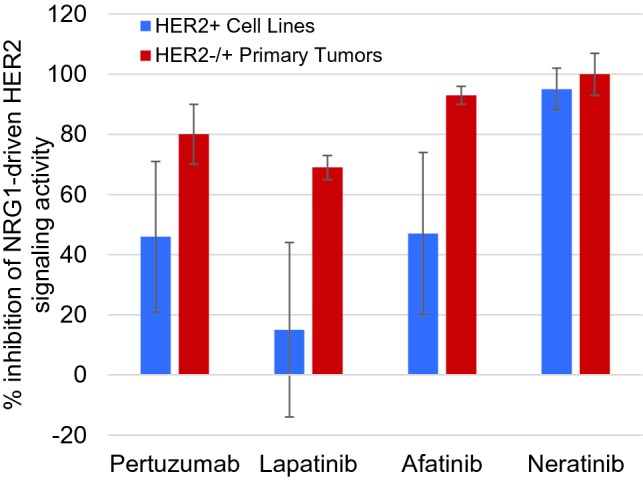
Comparison of inhibition of NRG1-driven HER2 signaling by HER2 targeted therapeutics. Pertuzumab (10 μg/mL), lapatinib (200 nM), afatinib (120 nM), or neratinib (500 nM) inhibition of NRG1-driven HER2 signaling is shown as the average of 9 HER2+ cell lines (SKBR3, EFM192A, ZR75-30, HCC202, HCC1954, HCC1569, MDA-MB361, BT474, AU565) or 7 HER2−/HER2s+ primary tumors (R69, R20, R160, R82, R95, R25, R71) performed with 2 technical replicates per biological sample. Single dose concentrations were selected for relative comparator purposes only where a maximal amount of the signaling was inhibited in at least one of the samples. Variances from 100% inhibition likely arise from ligand generated signaling that was not directly related to HER activity or where a sample had some level of drug paradoxical response (Claus et al. 2018)

HER2-negative primary tumor samples found to have abnormal HER2-driven signaling by the CELx HSF test (*n* = 7) and HER2+ cell lines (*n* = 9) were further evaluated to determine the amount of signaling initiated by the NRG1b agonist that could be inhibited by a HER2 mAb (pertuzumab) and three small molecule pan-HER receptor tyrosine kinase (RTK) inhibitors (lapatinib, neratinib, and afatinib) at therapeutic concentrations (Fig. 4). Changes in cell proliferation can be detected when an excess amount of a kinase inhibitor is applied due to less specific off target effects (Klaeger et al. 2017; Zhang et al. 2008). For example, the lapatinib data in the Cancer Cell Line Encyclopedia report many HER2+ cell lines with EC_50_ and IC_50_ values greater than 1 µM (Barretina et al. 2012). We have selected concentrations of drug that indicate a level of sensitivity and specificity appropriate for measuring on-target potency and efficacy ex vivo and for assessing an in vivo anti-tumor drug effect in an animal model. Each HER2-targeted drug inhibited as much or more of HER2-driven signaling in the HER2−/HSFs+ primary tumor cells than in the HER2+ cell lines. The HER2 drugs inhibited an average of at least 69% of the HER2-initiated signaling stimulated by NRG1 in the HER2-negative primary cell samples; the highest level of inhibition was found with the two irreversible covalent dual RTK inhibitors, afatinib and neratinib (Fig. 4). Such similarities in response range and pattern suggest similarity in underlying disease mechanisms.

### Mouse xenografts of a HER2−/HSFs+ cell line are sensitive to lapatinib but a HER2+ /HSFs- cell line is not

To support the utility of the CELx HSF Test results and demonstrate the ability of the test to identify HER2-negative cells that are responsive to HER2 targeted therapy, we next evaluated the efficacy of lapatinib on growth of xenografts of two human breast cancer cell lines. Cell lines were selected based on both extensive characterization of HER2 status and response in the CELx HSF assay (Supplemental Table 1). As demonstrated, the HER2+ cell line, HCC1954, showed normal HER2 signaling in the CELx HSF assay, while the HER2-negative cell line, BT483, showed normal HER2 protein expression but abnormally high HER2 signaling in the CELx HSF assay (Supplemental Table 1).

**Fig. 5:**
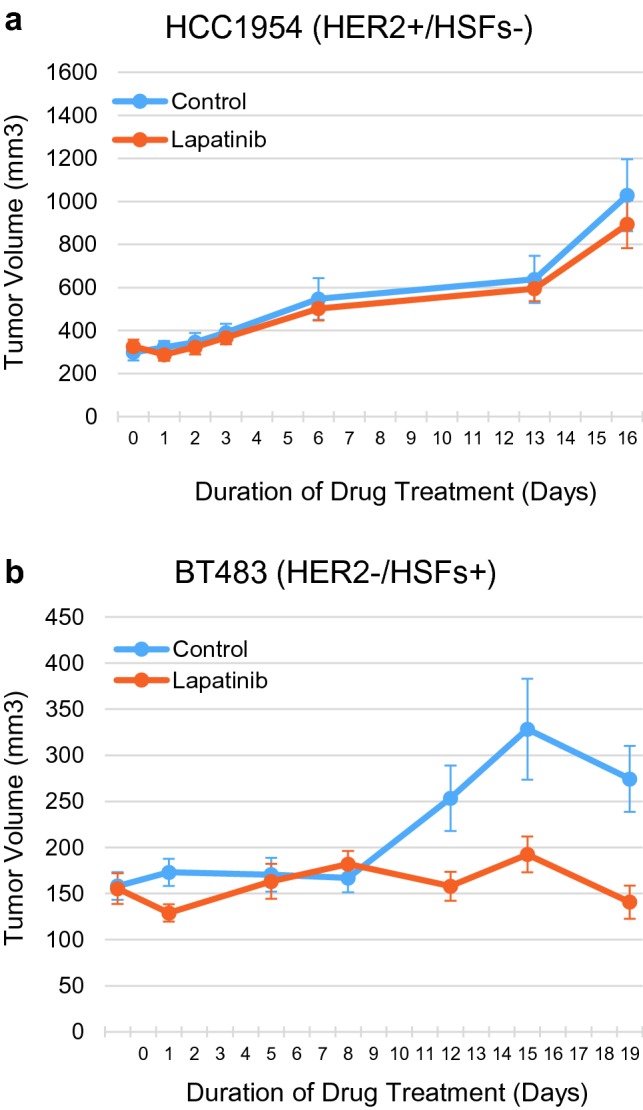
Lapatinib inhibits growth of a HER2- xenograft exhibiting abnormal HER2 signaling but not a HER2+ xenograft exhibiting normal HER2 signaling as assessed by the CELx HSF test. **a** Growth of HCC1954 cells in a xenograft model is not inhibited by the HER2-targeted therapeutic lapatinib. **b** Lapatinib suppresses growth of BT483-derived xenografts. Lapatinib administration was initiated when tumor volume was 150 mm^3^ (BT483) or 300 mm^3^ (HCC1954). Data are shown as average tumor volumes (*n* = 10 per group)

Administration of lapatinib did not inhibit tumor growth of xenografts of the HER2+ /HSFs- human breast cancer cell line (HCC1954; Fig. 5a). HCC1954 xenografts grew readily in the lapatinib treated arm. No significant differences in tumor size were observed between the lapatinib-treated cohort and the control cohort over the entire study duration (*P* = 0.285). Although HCC1954 is reported to have varying sensitivity to high concentrations of lapatinib in vitro (O’Brien 2010; O’Neill 2012; Luoh 2019), to the best of our knowledge this is the first study to assess sensitivity to lapatinib alone in a xenograft model of breast cancer. In contrast, HER2−/HSFs+ BT483 cells grew more slowly as xenografts, but lapatinib administration resulted in significant and sustained inhibition of tumor growth, which was apparent within 12 days of the onset of treatment (Fig. 5b). For the control group (*n* = 9), the average tumor size at the end of the treatment period was 1.73 times the size of the starting tumor volume. For the lapatinib-treated group (*n* = 10), the average tumor size at the end of the treatment period was 0.91 times the size of starting tumor volume. The treatment arm tumor change vs. the control arm tumor change (T/C ratio) was 0.52 (*P* = 0.01), a statistically significant difference. The results are consistent with our previous CELx Test findings that BT483 cells have abnormally high HER2 signaling activity despite having normally expressed HER2 (Supplemental Table 1; (Huang et al. 2017).

The efficacy of lapatinib in xenograft models of human breast cancer cell lines did not correlate with either HER2 expression or HER2 gene copy number (Supplemental Table 1), but rather correlated with abnormal HER2 signaling as measured by the CELx HSF assay. This data suggest that measuring HER2 pathway activation can be a better predictor of response to HER2 targeted therapeutics than HER2 protein expression or copy number and confirms the potential utility of the CELx HSF Test assay as a clinical tool to supplement HER2 testing for treatment decisions.

### Clinical parameters of 114 breast cancer patient sample set

To confirm the prevalence of abnormal HER2 signaling amongst HER2-negative tumor tissues, we used tissue from a cohort of 114 patients for CELx HSF testing, cutoff determination, and sub population analysis (Supplemental Table 2). Patient mean age was 58.6 years. The majority of tumors were classified as Stage II, but all stages were represented. Most tumors belonged to the ductal invasive/mixed or ductal invasive/lobular mixed histological subtypes and were estrogen receptor positive (ER+), with variable lymph node status. HER2-negative clinical status (ISH and/or DAKO IHC scores) was confirmed and provided by multiple clinical laboratories and indicated that all patients were clinically HER2-negative. Based on pathology reports, 13 tumors that were initially IHC equivocal were subsequently analyzed by HER2:CEP17 FISH. No patients with HER2 equivocal FISH scores were included in this study. The average of the HER2:CEP17 FISH ratio scores reported was 1.11, with one patient having the maximum score of 1.3. As such, there is no likelihood that patients in this cohort were considered for clinical treatment with a HER2-targeted drug.

Following cell colony establishment and growth, cell samples were prepared for testing in the CELx HSF Test assay. Flow cytometric analysis was performed on samples using excess material to confirm the ISH and/or IHC designation by the clinical site and to provide a standard central laboratory measurement across all samples (Supplemental Fig. 2 and Supplemental Table 4). The results of flow cytometric analysis were concordant with the standard clinical pathology test evaluations for HER2 that were provided for each specimen. All primary mammary epithelial tumor cell samples expressed a range of HER2 levels, as measured by flow cytometry, which remained within the normal spectrum of HER2 expression and below the expression level of the DAKO 1 + MDA175vii control cell line (Supplemental Fig. 2 and Supplemental Table 4).

### Identification and prevalence determination of a subset of HER2-negative patients with abnormal HER2 signaling

Having previously validated the ability of the CELx HSF test to identify cells with abnormal HER2 signaling (Huang et al. 2016), the present study aimed to determine a cutoff for the CELx HSF Test in this clinically HER2-negative population and to determine a prevalence rate of abnormal HER2 pathway signaling in a cohort of HER2-negative patients sufficiently large enough to be statistically well-powered. The CELx HSF Test was applied to the independent set of 114 tumors that were clinically HER2-negative to determine whether a sub-group of these samples had abnormal HER2 pathway signaling. The DAKO IHC clinical standard HER2+ breast cancer cell line (SKBr3) was included in each run as a positive control. Total CELx HSF HER2-dependent signals (NRG1b-induced and EGF-induced) were obtained for all primary tumor samples (Supplemental Table 4). The output of the CELx HSF test is recorded as signaling response units, and as previously reported (Huang et al. 2016), we employ a cutoff of 250 signaling response units as the threshold for abnormally active HER2 signaling in primary breast cancer cells.

CELx HSF test scores for the current data set (*n* = 114) were analyzed for consistency with the previously published smaller dataset (*n* = 34) and for goodness of fit to a multicomponent “mixture” model of HER2-negative tumor types with variable degrees of abnormal HER2-mediated signaling. We tested the current data set for compatibility with a separate cohort of patients using the Kolmogorov–Smirnov non-parametric two-sample test for identity of the distributions was used (Huang et al. 2016). The test statistic obtained indicated no significant difference between the CELx HSF scores of these two groups (*D* = 0.16642; *P* value of 0.4485), supporting the hypothesis that determinations from the smaller dataset will be concordant with the current, larger dataset but with greater statistical power. Next, a normal mixture model was fitted to the 114-patient data set using the *normalmixEM* procedure in the R package mixtools. Two runs of the *normalmixEM* procedure were performed, fitting 2 and 3 components, along with a baseline single-component normal distribution model (Table 1).

**Table 1:** “Goodness of Fit” for multicomponent mixture models of HSF scores of HER2-negative tumor cells

Components analysis						
# of components	1	2		3		
Subset	A	A	B	A	B	C
Mean	145.7	25.1	237.6	4.9	104.4	376.6
St. deviation	156.6	27.8	150.8	6.3	60.1	114.1
Proportion	1.00	0.43	0.57	0.26	0.49	0.25
Loglikelihood	− 737.4	− 704.0		− 676.7		
Chi-squared		66.92		52.24		
*P* value		1.9e−14		2.7e−11		

**Fig. 6:**
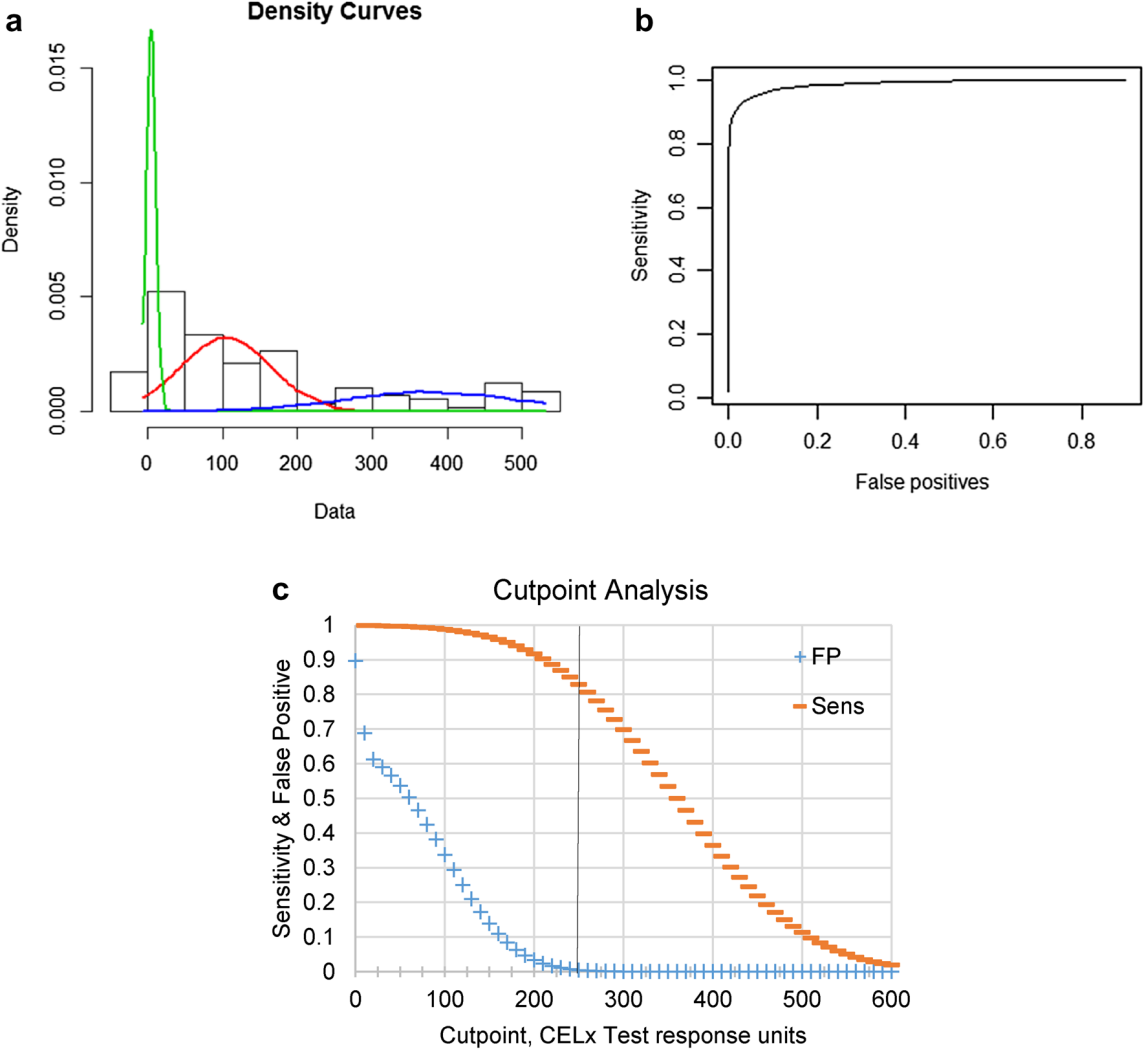
Establishment of a cutoff for abnormal HER2 signaling in the CELx HSF Test.** a** Three-component normal mixture fit superimposed on a histogram of the individual CELx HSF scores (*x *axis) for HER2-negative tumors (*n* = 114). Green, red, and blue curves represent subsets *A*, *B*, and *C* (respectively) of the three components analysis detailed in Table 1. **b** Inferred ROC curve for component three vs. a composite of components one and two. **c** Relationships of sensitivity (Sens, false negatives, < 15%) and false positives (FP = 0) to potential CELx HSF Test cutpoints derived from the ROC analysis. The vertical line is the selected cutpoint at 250 CELx Test signaling units

Statistical analysis of multicomponent mixture models, where components A, B, and C, respectively, refer to the green, red, and blue density curves of CELx HSF test scores for 114 tumors shown in Fig. 6a

Formal significance testing of the likelihood that two or three different patient populations characterized by CELx HSF test scores (components) exist within the tumor data set showed that a two-component mixture model is a much better fit than a common normal distribution of the 114 patient test results, and a three-component mixture is better than either a two-component mixture or a normal distribution (Table 2).

**Table 2:** Likelihood ratio test of a two or three component mixture model of HSF scores of HER2-negative tumor cells

Test of fits	Chi-squared	*P* value
2 vs 1	66.92	1.8e−14
3 vs 2	54.59	8.7e−12

Statistical analysis using a likelihood ratio test reveals that three-component mixture model significantly improves goodness-of-fit compared to a two-component mixture model, where components refer to density curves of CELx HSF test scores shown in Fig. 6a

When the three-component fit is superimposed on a histogram of the individual CELx HSF test scores for the HER2-negative tumors, the right-most fitted component three (Fig. 6a, blue curve) comprises 25% of the population (Table 1, proportion of population C) and has a mean and standard deviation of 377 and 114, respectively. The values for the fitting of this data were used to construct a receiver operating characteristic (ROC) curve (Fig. 6b) for distinguishing the third population component (the abnormal HER2 signalers) from the next putative normal HER2 signalers in a 1:2 mixture of the first and second components of the three-component fit model. The inferred ROC analysis for estimating sensitivity (true positive, false negative) and specificity (false positive, true negative) demonstrates that our ability to detect a true positive (sensitivity), a HER2-negative tumor with abnormal HER2 signaling, begins to decrease upon approaching a cutoff of approximately 200 signaling response units, with only a 10% loss in sensitivity at a cutoff of 250 signaling response units (Fig. 6c). Likewise, the likelihood of determining something is a false positive (i.e., mistakenly characterizing a HER2-negative tumor as having abnormal HER2 signaling) approaches a minimum at a cutoff of 200 signaling response units and is virtually zero by the cutoff of 250 signaling response units. These analyses support the use of a CELx HSF cutoff score of 250 signaling response units as suitable for identifying approximately 90% of patients with abnormal HER2 signaling with minimal or no false positives.

Table 3 shows the percent of clinically HER2-negative patients whose tumors have > 250 signaling response units in the CELx HSF Test and qualify as exhibiting abnormal HER2 signaling function, which we refer to as HER2−/HSFs+. Of the HER2-negative breast tumor cell samples tested, 27 of 114 patients (24%; 95% CI 17%–32%) exhibited HER2 signaling activity that was determined to be above the cutoff value and, therefore, was characterized as abnormally high.

**Table 3:** CELx HSF test summary results of clinically HER2-negative patient tumor samples

Characteristic	No. of patients	No. abnormal signaling patients	% of category
Total patients	114	27	24
Stage			
I	23	5	22
II	62	11	18
III	25	9	36
IV	4	2	50
Estrogen receptor status			
ER+	96	24	25
ER−	18	3	17

## Discussion

Using the CELx HER2 Signaling Function Test, we demonstrated that the current standard of care therapies for HER2 positive patients (trastuzumab plus pertuzumab) inhibited a similar percentage of HER2 signaling in HER2-negative cells as that found in HER2+ cells. Furthermore, four HER2 signal inhibitors (pertuzumab, lapatinib, neratinib, or afatinib) were also each effective at inhibiting HER2-initiated signaling in the HER2−/HSFs+ primary tumor cells and in the HER2+ /HSFs+ cell lines, suggesting that the underlying disease mechanisms of abnormal HER2 signaling (HSFs+) may be similar regardless of whether the tumor cell is categorized as HER2-negative or HER2+ by IHC or FISH. Extending this work to mouse xenograft studies, we confirmed that a HER2-negative cell line that was HSFs+ responded to lapatinib in vivo. Lapatinib administration was shown to reduce the rate of tumor growth only in the animals with tumors derived from the HER2-negative/HSFs+ cell line, and not the HER2+ /HSFs− cell line. Taken together, these results provide an in vivo demonstration that efficacy of HER2 targeted therapies correlate with the real-time CELx Test measure of HER2 signaling activity and not with HER2 expression or gene copy number.

The present study expanded on initial development (Huang et al. 2016) by testing 114 HER2-negative patient tumor samples to confirm that a clinically significant subset of HER2−/HSFs+ tumors exists and to establish a robust determination of the cutoff value for normal versus abnormal HER2 signaling in breast cancer patient cells. Statistical analyses of the CELx HSF test results for the 114 patient tumor samples determined that a sub-group of tumors with very high HER2 signaling activity existed and further confirmed that the appropriate cutoff value between abnormal and normal was 250 signaling response units. This cutoff is predicted to result in minimal false positives while still identifying almost 90% of those with abnormal HER2 signaling. Using this cutoff value, the prevalence of HER2-negative tumors in the 114-tumor dataset with abnormally high signaling activity was also determined to be approximately 20%. No associations or correlative patterns were found between CELx test signaling responses and patient pathologic information.

Current selection of patients for HER2-targeted therapies includes measurements of HER2 protein levels and/or HER2 gene copy number in tumor biopsies (Wolff et al. 2013). For HER2-postive patients receiving anti-HER2 therapies, response rates are typically only 30–35%, suggesting that using HER2 status as a patient selection biomarker generates a high rate of false positive diagnoses. Additionally, a subset of HER2-negative patients treated with trastuzumab in the NSABP B-31 and NCCTG N9831 clinical trials responded similarly as HER2+ patients, evidencing the false negative rates for current biomarkers.

Previously, statistical data simulations have been performed that assess the ability of a clinical trial to identify a treatment difference in an unselected patient population. When the survival outcome was modeled for the case where 25% of the patient population would respond to a targeted therapeutic versus placebo, the survival curves converge such that the benefit of the targeted therapy would be obscured (Pegram et al. 2005). This effect may explain why a subpopulation of HSFs+ responders was not observed in trials such as the NSABP B-47, which was designed to evaluate the addition of trastuzumab to the standard of care chemotherapy in women with tumors that were IHC 1+ or 2+ . If we assume a similar prevalence of abnormal signal function in the HER 2− low group of NSABP B-47, we would not have predicted success of the NSABP B-47 trial without screening for HER2 signaling dysfunction to select the responsive patient population.

The clinical results to date suggest that neither HER2 protein levels nor gene copy number are sufficient to explain treatment response. The results presented here demonstrate the importance of identifying the high level of signaling dysfunction as the disease characteristic whose attenuation leads to drug efficacy and potency. Treatment directed at patients with normal levels of HER2 signaling, regardless of the amount of protein, is not expected to affect disease status.

Cancer has been described as a loss of the adaptive mechanism that regulates cell response to change in input stimulus and its subsequent return to the pre-stimulated response level, even when the change in input persists (Ferrell 2016). Cytokine signaling through RTKs such as EGFR and HER3 is described as involving multiple adaptive mechanisms at many different molecular sites, which may explain the challenge in using a point in time protein biomarker to monitor signaling activity or response to therapy. In perfect and near perfect adaptation of cell signaling, there have been defined a small number of elementary signaling motifs that can describe response termination (Ferrell 2016; Ma et al. 2009). However, there may be a very large number of ways in which adaptation may be disrupted at the molecular level that would be difficult to detect or predict without dynamic data capable of characterizing the effect of disruption of control of all epigenetic, transcriptional, translational, and post-translational processes. While the CELx test method does not directly identify the exact molecular cause(s) of a patient’s cancer, the signaling response output reflects a quantification of the total activity associated with the cell signaling system. The CELx test output summarizes the outcome of the large number of permutations of the signaling system and system adaptations in the context of the patient’s normal sequence variants as well as any abnormal sequence variants (Huang et al. 2016). If the variants that are present prevent cellular adaptive mechanisms from functioning normally, then hyperactivity of the signaling system may occur, which is what the test output is designed to detect. The CELx test essentially identifies patients with tumors where the normal adaptive response has become ineffective and verifies that application of targeted antagonists to that patient can effectively attenuate their dysfunctional signaling.

Collectively, the results presented here demonstrate that the CELx HSF Test is a reliable, extensively characterized biomarker assay capable of identifying dynamic HER2-initiated signaling dysfunction in tumor cells from HER2-negative breast cancer patients. With the CELx HSF test, a new sub-group of HER2-negative breast cancer patients with abnormal HER2 signaling (HER2−/HSFs+) has been identified that may receive benefit from approved HER2-targeted therapies. Given that the patient specimens and supporting information were deidentified prior to arrival and analysis, it was not possible to correlate the results of the CELx HSF test with treatment response or patient outcomes in this study. To validate whether this new sub-group of HER2-negative breast cancer patients may benefit from the addition of small molecule HER2 inhibitors or HER2 mAbs to current therapeutic regimens, several clinical trials have been initiated.

In one single-arm open-label study sponsored by the NSABP Foundation (NSABP FB-12/FACT-1), early stage HER2-negative breast patients identified by the CELx HSF test to have abnormal HER2 signaling will receive the same standard of care therapy regimen early stage HER2+ breast cancer patients (chemotherapy plus trastuzumab plus pertuzumab) receive.

In another single-arm open-label study sponsored by the University of Tennessee West Cancer Center (FACT-2), early stage triple-negative breast cancer patients with abnormal HER2 signaling will receive the irreversible pan-HER inhibitor neratinib in combination with chemotherapy. In both trials, the primary endpoint is pathological complete response. The rationale for conducting these trials in an early stage neoadjuvant setting is based on the fact that early stage HER2+ breast cancer patients treated with neoadjuvant anti-HER2 therapies have been found to have significantly higher rates of pathological complete response than early stage HER2-negative patients receiving standard of care neoadjuvant chemotherapies (Cortazar et al. 2014).

## Conclusion

An analytically validated live cell test that measures dynamic HER2-initiated signaling activity in tumor cells ex vivo can be used to identify a subset of HER2-negative breast cancer patients responsive to HER2 therapies. This test is currently being studied in interventional clinical trial settings.

## Electronic supplementary material

Below is the link to the electronic supplementary material.

Supplementary file1 (DOCX 15919 kb)

## Abbreviations

CAS: Cellular adhesion signal
EGF: Epidermal growth factor
ER: Estrogen receptor
HER2: Human epidermal growth factor receptor 2
HSF: HER2 signaling function
HSFs+: Abnormal CELx HSF Test Score
HSFs-: Normal CELx HSF Test Score
IHC: Immunohistochemistry
ISH: In situ hybridization
NCCTG: North Central Cancer Treatment Group
NRG1: Neuregulin 1
NSABP: National surgical adjuvant breast and bowel project
ROC: Receiver operating characteristic
RTK: Receptor tyrosine kinase
