# Supplementary material for: New HER2-negative breast cancer subtype responsive to anti-HER2 therapy identified
Source: J Cancer Res Clin Oncol. 2020 Feb 8;146(3):605–19. doi: 10.1007/s00432-020-03144-7 (PMC7039866; doi:10.1007/s00432-020-03144-7)
Supplement: Supplementary file 1 — Supplementary file1 (DOCX 15919 kb) [file 432_2020_3144_MOESM1_ESM.docx]

**Supplemental Information**


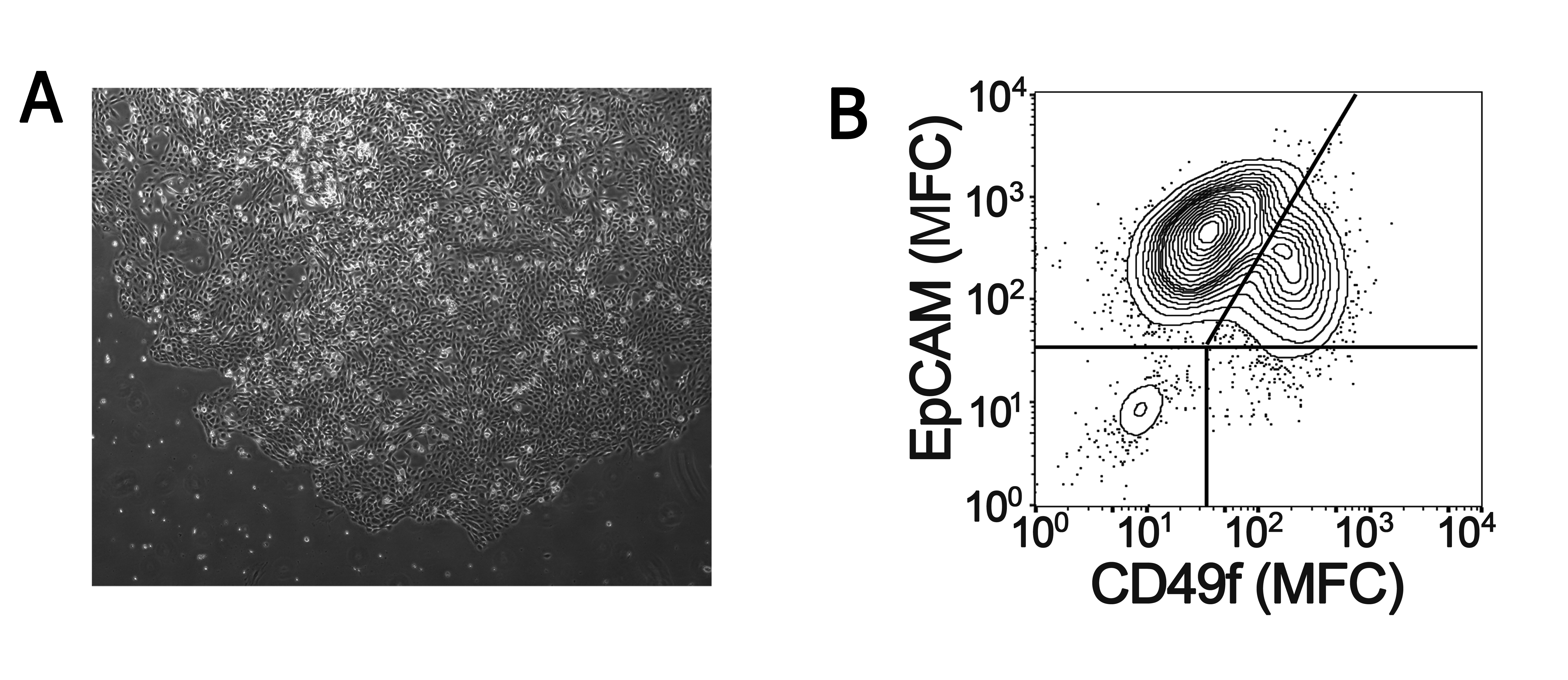


B


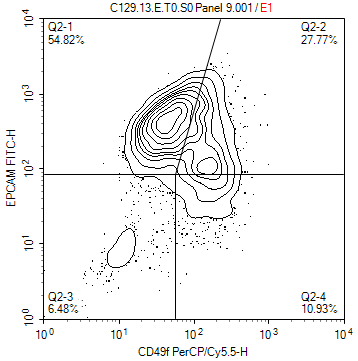

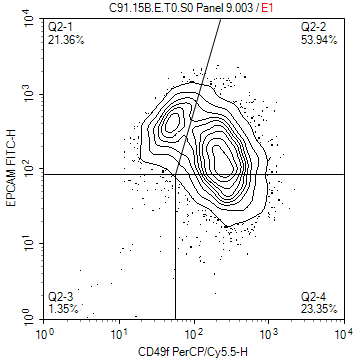


C91

C129

CD49f

CD49f

EpCAM

EpCAM

**Supplemental Figure 1: Cultured tumor cells isolated from patient biopsies are phenotypically epithelial.
(A)** Representative culture of primary cells from a digested tumor biopsy at day 9 of culture. **(B)** Flow cytometric analysis of the epithelial luminal biomarker EpCAM and the basal/progenitor biomarker CD49f expression level on the tumor primary cells analyzed in Figure 1 at the time of CELx HSF test.

**Supplemental Figure 2**. FACS analysis of HER2 expression confirms the HER2- status designated by IHC and/or ISH. 114 patient samples were analyzed for Her2 expression using flow cytometry as described in the methods. The mean fluorescence intensity of the samples were compared to three cell lines used as standards in the DAKO HercepTest. (SKBR3, DAKO 3+, MDA175vii 2+, MDA231 0)


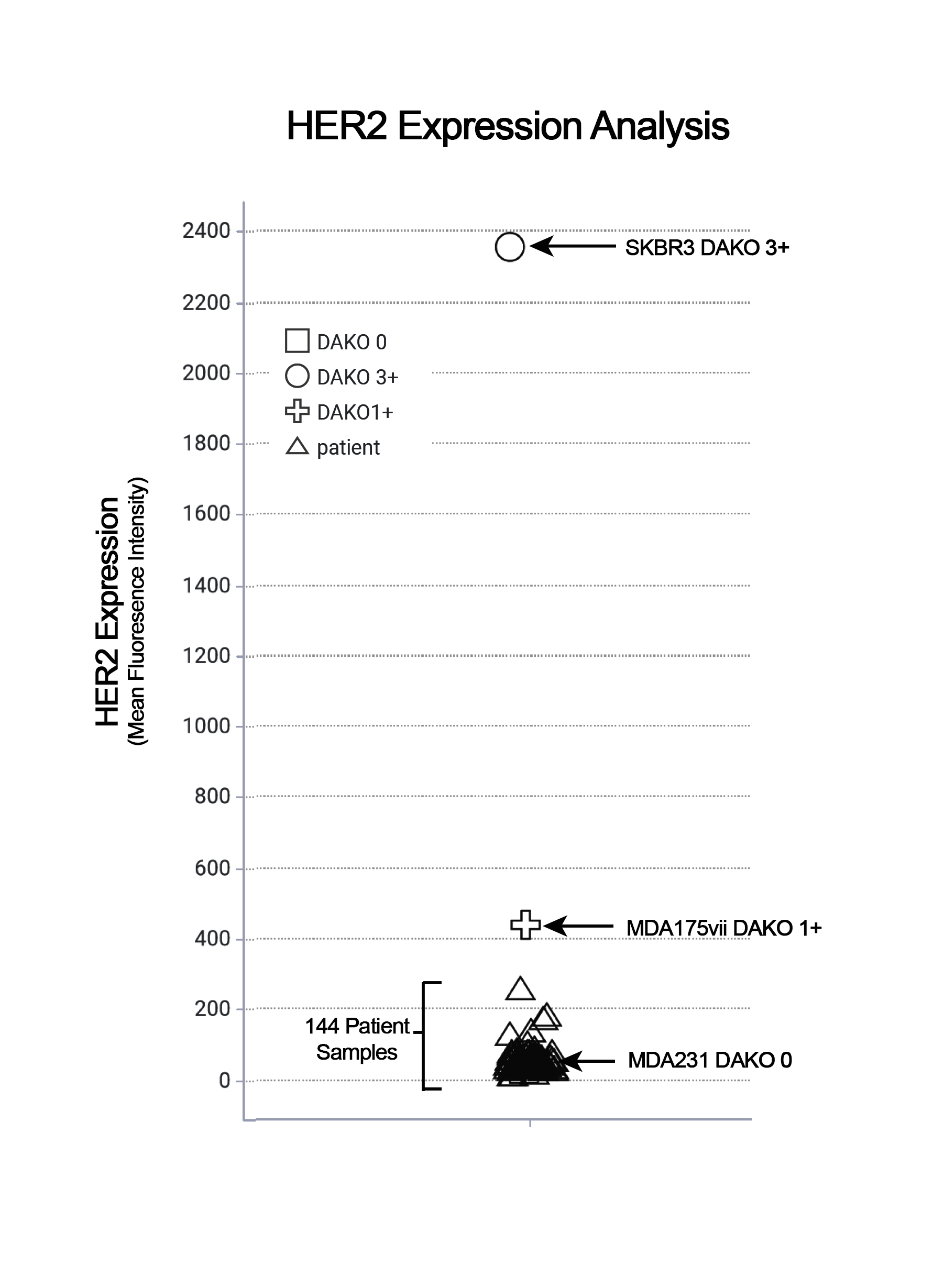


114 Patient

Samples

| **Supplemental Table 1. Characteristics of HCC1954 and BT483 cells** | | |
| --- | --- | --- |
| Cell Line | HCC1954 | BT483 |
| HER2 status | HER2+ | HER2- |
| Celcuity HER2 protein level (MFC*) | 4009 | 441 |
| CCLE HER2 mRNA level (Log2) | 12.6 | 9.6 |
| CCLE HER2 gene copy (Log2) | 3.46 | 0.17 |
| CELx HSF Test results | HSF_S_- | HSF_S_+ |
| CELx HSF score (cutoff = 250 units) | 88 | 454 |

* MFC- Mean Fluorescence Channel

**Supplemental Table 2. Breast Cancer Patient Population Characteristics**

| Characteristic | No. of Patients | % |
| --- | --- | --- |
| Total Patients | 114 |  |
| Age, years |  |  |
| Mean | 58.6 |  |
| Range | 36-85 |  |
| Stage |  |  |
| I | 23 | 20 |
| II | 62 | 54 |
| III | 24 | 22 |
| IV | 4 | 4 |
| Histology |  |  |
| DCIS only | 0 | 0 |
| Invasive only | 24 | 21 |
| Invasive Ductal/DCIS mixed | 55 | 48 |
| Lobular/Other | 35 | 31 |
| Lymph Node Status |  |  |
| Metastatic | 56 | 49 |
| Non-Metastatic | 46 | 40 |
| pNX or NA* | 12 | 11 |
| Estrogen Receptor Status | |  |
| ER+ | 96 | 84 |
| ER- | 18 | 16 |
| HER2 FISH/IHC Scores |  |  |
| Negative/0/1+/FISH not amplified** | 101 | 89 |
| IHC2+/FISH not amplified | 13 | 11 |

* Information not available

** Some hospitals report only FISH score

**Supplemental Table 3. Antibodies used in flow cytometric analysis**

| Antigen | Conjugate | Clone | Source |
| --- | --- | --- | --- |
| CD10 | Allophycocyanin | HL10a | BioLegend |
| CD49f | PerCP-eFluor710 | eBioGoH3 | eBioScience |
| Claudin 4 | Phycoerythrin | 382321 | R&D Systems |
| EPCAM | AlexaFluor488 | MH99 | eBioScience |
| HER2 | Phycoerythrin | 24D2 | Biolegend |
| PRG | eFluor660 | KMC912 | eBioScience |
